# Psychotherapy versus usual care in pediatric migraine and tension-type headache: a single-blind controlled pilot study

**DOI:** 10.1186/1824-7288-40-6

**Published:** 2014-01-20

**Authors:** Umberto Balottin, Matteo Ferri, Michela Racca, Maura Rossi, Giorgio Rossi, Ettore Beghi, Matteo Chiappedi, Cristiano Termine

**Affiliations:** 1 Department of Child Neurology and Psychiatry, C. Mondino National Neurological Institute, and University of Pavia, Pavia, Italy; 2 Child Neuropsychiatry Unit, Department of Clinical and Biological Sciences, University of Insubria and “Macchi Foundation”, Varese, Italy; 3 Department of Child Neurology and Psychiatry, C. Mondino National Neurological Institute, Pavia, Italy; 4 Laboratory of Neurological Disorders, IRCCS – Institute for Pharmacological Research “Mario Negri”, Milano, Italy

## Abstract

**Background:**

Despite growing interest in psychotherapy in child and adolescent headache, efficacy studies in this research field have focused mainly on cognitive-behavioral therapies. Whereas relaxation and cognitive-behavioral techniques, in particular, have been found to reduce the intensity and frequency of headache in children and adolescents, data on psychodynamic psychotherapy in this population are lacking.

Our aim was to explore the effectiveness of a brief psychodynamic psychotherapy program in the treatment of idiopathic headache in childhood and adolescence.

**Methods:**

Thirty-three newly diagnosed idiopathic headache sufferers aged 6–18 years, consecutively referred to our outpatient services, were randomized to receive either a brief cycle of psychodynamic psychotherapy (eight sessions administered at two-week intervals) or usual care (clinical interview, neurological examination, counselling, symptomatic therapy).

The two groups were evaluated at baseline (T0) and at six months (T1) to be assessed for headache characteristics (i.e. frequency, intensity and duration), quality of life (i.e. the EuroQoL score), patient’s global health status (i.e. the Clinical Global Impression score), and emotional-behavioral symptoms (i.e. Child Behavior Checklist scores).

**Results:**

The two groups were fairly similar with reference to the main demographic and clinical variables. The T0/T1 comparison showed a statistically significant improvement in headache frequency (p = 0.005), intensity (p < 0.001) and duration (p = 0.002), a statistically significant improvement in the CGI score (p = 0.018), and a borderline improvement in the EuroQoL score (p = 0.053) in the group receiving psychotherapy.

**Conclusions:**

According to our pilot findings, a brief psychodynamic psychotherapy program may be more effective than usual care in children and adolescents with idiopathic headache.

## Background

Idiopathic headache is a common and disabling condition in children and adolescents and the possible role of psychological factors in its onset and course is still debated [1,2]. Controlled studies have revealed a remarkable association of depression [3,4] and anxiety [4-6] with headache, especially in girls [7]. In a meta-analytic study, children and adolescents with migraine and tension-type headache showed more psychopathological symptoms than did healthy controls [8].

Three main hypotheses have been advanced to explain the association between psychopathology and headache: 1) the existence of a bidirectional causality between psychological disorders and headache; 2) the presence of common etiological factors; and 3) the presence of an interaction between psychological and somatic factors [9-11]. Moreover, children with headache have a greater risk of developing psychological disorders in adulthood compared with their healthy peers, and early intervention in these subjects may reduce this risk significantly [12].

Despite a growing interest in psychotherapy in child and adolescent headache, efficacy studies in this research field have focused mainly on relaxation and cognitive-behavioural therapies, which have been demonstrated to be effective in reducing the intensity and frequency of the attacks [2]. Although short-term psychodynamic psychotherapy has been suggested to be effective in specific disorders [13,14], observations regarding headache patients treated with the psychodynamic approach [15-17] are, at present, only anecdotal. Nevertheless, this approach is widely applied in headache in several countries, including Italy.

Taking these considerations as our starting point, we conducted a controlled, randomized pilot study in which we compared a short-term psychodynamic psychotherapy program with usual care for the treatment of children and adolescents with idiopathic headache.

Our primary aim was the improvement of headache characteristics (i.e. frequency, intensity, duration), quality of life ratings and behavioural and social skills in the short-term.

## Methods

Eligible patients were children and adolescents with headache consecutively referred, for a first consultation, to the child neuropsychiatry units of the Universities of Varese and Pavia, Northern Italy. The criteria for inclusion in the study were: age between 6 and 18 years; diagnosis of idiopathic headache (migraine without aura - MO, migraine with aura - MA, tension-type headache - TTH) according to the original criteria of the International Headache Society classification [18]; normal neurological examination and absence of major psychiatric or neurological comorbidities; the presence of at least one headache attack in the previous month; no current prophylactic therapy.

At the first consultation (i.e., at recruitment, T0) the physician (i.e. a child neuropsychiatrist trained in child and adolescent psychiatry and psychotherapy) evaluated the characteristics of the patient’s headache (frequency, intensity, duration) over the past six months and gave the patient a headache diary, to be completed prospectively throughout the study. The diary was used to collect information on headache characteristics, coded on a monthly basis, and on drug intake for pain relief.

At a second consultation (T0), one month later, we verified the patients’ fulfilment of the inclusion/exclusion criteria using a clinical interview specifically designed to evaluate (and exclude) major comorbid psychiatric symptoms. After providing a detailed explanation of the study rationale and aims, we obtained informed consent from the patients and parents in accordance with the requirements of the ethics committees of the two participating centres. Data were then collected on the patient’s global health status (using the Clinical Global Impression (CGI) scale [19]), the patient’s quality of life (using EuroQoL [20]), and the parents’ perceptions of the patient’s behavioural problems (using the Child Behavior Checklist (CBCL) 4–18 [21]).

Separate randomization lists in sealed envelopes, one for each centre, were prepared by an independent centre (the Mario Negri Institute, Milan).

The eligible subjects releasing an informed consent were randomly assigned to the experimental group or the control group. The experimental group was subsequently divided in two subgroups on the basis of the patient’s willingness, or not, to be treated in an individual setting: the individual-setting group (i.e., just the patient) (n = 9) and the family-setting group (i.e., the patient and both parents) (n = 8).

In the experimental group, the short-term psychodynamic psychotherapy program consisted of eight sessions of psychotherapy, which took place at two-week intervals. As above indicated, this treatment model was applied in an individual or a family setting.

These sessions were administered by expert child and adolescent psychotherapists according to a protocol that, focusing on the separation-individuation process, had been developed on the basis of a short psychotherapy manual [22]. In the first two sessions the therapist explored the patient’s relationships with siblings and adults (the parents in particular), focusing mainly on conflicts relating to the separation-individuation process [22]. In the subsequent sessions the therapist, together with the patient, explored more deeply the conflicts the therapist felt would be sensitive to the intervention. The patient, through empathic identification with the therapist (mirror identification), was helped to reach a progressively more adaptive understanding of his/her difficulties. A final interview, with the patient alone (individual-setting group) or with the patient and both parents (family-setting group), concluded the treatment. In the latter case (eight sessions conducted with the parents and the patient), intrafamilial dynamics were explored as potentially causative of the occurrence and maintenance of the patient’s headache. The aims of the therapy were to identify the family’s theory of headache, and the significance of the symptom within the family dynamics, in order to help the family better express and manage negative and intense feelings and conflicts, and allow the parents to fulfil their role, protecting and supporting their child. We chose this study design because it closely reflects the short-term psychodynamic psychotherapeutic approaches usually used in our clinical practice.

The patients in the control group were managed in accordance to clinical practice (usual care) (clinical interview with the patient and the parents every two months, headache diary assessment, neurological examination, counselling, and symptomatic therapy if necessary), always with the physician who had conducted the initial consultation. No patient received prophylactic medications, either because this was not considered useful by the assessing physician (patient with 3 or less headache attacks per months) or refused by the parents (other patients). Usual care was the same in the two participating institution, whose staff has the same educational and scientific background.

All patients were re-evaluated six months after admission a neuropsychiatrist blinded to the approach used (T1). Data on headache characteristics, the patient’s global health status (CGI), the patient’s quality of life (EuroQoL), and the parents’ perceptions of the patient’s behavioral problems (CBCL) 4–18) were again collected.

Statistical analysis was performed to compare headache features (i.e., frequency, duration and intensity of attacks) and CGI, EuroQoL and CBCL scores at recruitment (T0) and after six months (T1).

Frequency of headache attacks was coded as: 0 if no attacks were reported, 1 if attacks were fewer than 1/month, 2 if they were 1-3/month, or 3 in the case of ≥4 attacks/month. Intensity was coded on a clinical basis as: 0 if no attacks were reported, 1 if attacks were mild, 2 if they were moderate, or 3 if they were severe. Duration was coded as: 0 if no attacks were reported, 1 if they lasted less than 1 hour, 2 if they lasted 1–3 hours, or 3 if they lasted more than 3 hours. The T0-T1 difference in the clinical parameters describing headache frequency, intensity and duration was categorized on the basis of a five-level categorical variable: worsening (T0-T1 < 0), no change (T0-T1 = 0), mild improvement (T0-T1 = 1), moderate improvement (T0-T1 = 2), marked improvement (T0-T1 = 3).

Data were compared using contingency tables, and a Chi-square test (Pearson’s or Mantel-Haenszel’s, as appropriate) was used for analysis of categorical variables.

The EuroQol and CBCL scores were expressed as mean (±SD) values to assess the comparability of the two treatment groups at baseline and as mean differences in T0-T1 comparisons. Quantitative variables were analyzed using the Kolmogorov-Smirnov normality test. Repeated measures analysis of variance (ANOVA) was subsequently applied.

In all the statistical comparisons two-tailed tests were applied and p = 0.05 was taken as the cut-off value for statistical significance. IBM SPSS Statistics software version 19 for Windows was used for the analysis. As this was a pilot study, we decided not to pre-determine the sample size nor to reset the level of significance, adjusting for multiple comparisons.

The study was approved by the Ethical Committees of both participating institutions (i.e. C. Mondino National Neurological Institute (Pavia, Italy) and Macchi Foundation (Varese, Italy).

## Results

**Figure 1:**
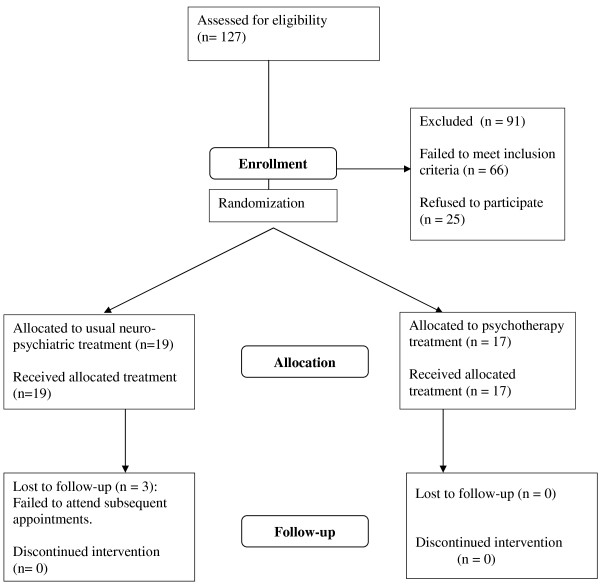
Study flow chart.

We examined a total of 127 consecutively referred headache patients: 36 (28.3%) fulfilled all the inclusion criteria and were admitted to the study. Of the 91 excluded patients, seven (7.7%) were younger than 6 years, 15 (16.5%) had a secondary headache and/or abnormalities in the neurological examination, four (4.4%) were taking prophylactic drugs, 21 (23.1%) did not fulfil the IHS criteria for MO, MA, or TTH, 19 (20.1%) had not experienced a headache attack during the previous month, and 25 (27.5%) did not give their informed consent (see Figure 1).

The sample comprised 17 males and 19 females aged between 6 and 18 years (mean age 9.67, SD 2.39). Seventeen patients were randomly assigned to the experimental group, and submitted to the short-term psychodynamic psychotherapy program in an individual (n = 9) or family setting (n = 8). Nineteen patients were randomized to the control group. All the patients (17/17) in the experimental group and 16/19 of those in the control group completed the study: three controls failed to attend subsequent follow-up appointments after the initial consultation (it was not possible to reach them on the phone to explore the reason for this).

Comparison of the experimental and the control group at T0 did not reveal significant differences in the main demographic and clinical characteristics, with the exception of a stronger intensity of attacks (Table 1) and a higher score on the Activities subscale of the CBCL in the experimental group (Table 2). At T0, the mean CBCL scores in the entire study sample (cases and controls) were all within normal limits.

**Table 1:** Baseline demographic and clinical characteristics of the cases and controls

	**Cases**	**Controls**	**p-value**
**N**	17	16	
**AGE** in years (mean ± SD)	9.18. ± 2.07	10.20 ± 2.72	0.227
**SEX**			
Males	9 (52.9%)	4 (25%)	0.101
Females	8 (47.1%)	12 (75%)	
**DIAGNOSIS**			
Tension-type headache	9 (52,9%)	7 (43,8%)	0.543
Migraine without aura	8 (47.1%)	8 (50%)	
Migraine with aura	0 (0%)	1 (6.3%)	
**Frequency of attacks**			
No attacks	0 (0%)	0 (0%)	0.109
<1/month	1 (5.9%)	0 (0%)	
1-3 /month	3 (17.6%)	8 (50%)	
≥4 month	13 (76.5)	8 (50%)	
**Duration of attacks**			
No attacks	0 (0%)	0 (0%)	0.219
<1 h	5 (29.4%)	1 (6.3%)	
1-3 h	5 (29.4%)	7 (43.8%)	
> 3 h	7 (41.2%)	8 (50%)	
**Intensity of attacks**			
No attacks	0 (0%)	0 (0%)	0.012
Mild	0 (0%)	5 (31.3%)	
Moderate	7 (41.2%)	8 (50%)	
Severe	10 (58.8%)	3 (18.8%)	
**CGI score**			
2	4 (23.5%)	5 (31.3%)	0.053
3	8 (47.1%)	1 (6.3%)	
4	5 (29.4%)	9 (56.3%)	
5	0 (0%)	1 (3.3%)	

**Table 2:** EuroQOL and CBCL six months after recruitment in the experimental and the control group:mean T0-T1 differences

	**Cases**		**Controls**		**p-value**		
	**T0**	**T1**	**T0**	**T1**	**W**	**B**	**I**
EuroQoL (*)	72.71 ± 18.81	86.29 ± 15.84	62.14 ± 10.14	63.86 ± 9.40	**0.021**	**0.000**	0.068
**CBCL**							
TOTAL (**)	55.07 ± 8.60	52.40 ± 8.58	53.79 ± 12.78	53.00 ± 13.28	0.259	0.929	0.535
INTERNALIZING (**)	61.80 ± 9.13	56.73 ± 10.80	54.50 ± 14.17	55.71 ± 14.57	0.410	0.301	0.184
EXTERNALIZING (**)	44.87 ± 11.00	47.27 ± 6.49	53.86 ± 11.25	48.64 ± 11.57	0.563	0.090	0.125
Withdrawn (**)	55.67 ± 9.03	55.20 ± 6.59	57.07 ± 7.91	57.21 ± 11.21	0.922	0.553	0.853
Somatic complaints (**)	68.07 ± 4.83	62.47 ± 7.53	65.57 ± 8.05	61.79 ± 8.08	**0.001**	0.504	0.492
Anxious/depressed (**)	58.87 ± 9.25	56.67 ± 6.50	58.36 ± 13.79	57.79 ± 12.85	0.576	0.925	0.742
Social problems (**)	54.33 ± 6.47	52.80 ± 3.63	56.64 ± 8.31	54.29 ± 6.41	0.153	0.344	0.758
Thought problems (**)	53.87 ± 4.70	54.87 ± 5.83	54.36 ± 6.71	55.14 ± 7.97	0.226	0.866	0.883
Attention problems (**)	56.20 ± 5.07	55.07 ± 4.88	57.00 ± 8.64	55.36 ± 6.83	0.240	0.799	0.827
Delinquent behavior (**)	53.20 ± 4.87	52.53 ± 4.05	53.36 ± 5.65	53.14 ± 6.29	0.719	0.804	0.853
Aggressive behavior (**)	52.20 ± 3.63	51.47 ± 3.04	53.43 ± 5.50	54.79 ± 8.55	0.812	0.166	0.429
Activities (*)	38.73 ± 6.67	40.20 ± 7.04	35.21 ± 7.15	33.64 ± 7.59	0.972	**0.029**	0.318
Social (*)	41.87 ± 8.11	43.00 ± 7.67	37.93 ± 8.36	43.14 ± 7.72	0.064	0.447	0.225
School (*)	50.00 ± 5.11	50.13 ± 4.72	76.07 ± 5.53	47.14 ± 9.35	0.614	0.105	0.695
Total competences (*)	39.73 ± 5.69	41.47 ± 6.73	42.00 ± 9.04	37.71 ± 7.33	0.298	0.761	**0.019**

*higher scores indicate a better health status.

**lower scores indicate a better health status.

W:within-groups factor (T0/T1).

B:between-groups factor (cases/controls).

I:W-B interaction.

Significant p-values are in bold.

The T0-T1 comparisons in the two treatment arms showed greater improvement in the experimental group in headache frequency (p = 0.005), intensity (p < 0.001) and duration (p = 0.002) (Table 3). These results were confirmed by the imputation of marked improvement in the three controls who dropped out of the study (Table 4). For frequency of attacks, a significant difference was retained only when mild improvement was also included.

**Table 3:** Main headache characteristics six months after recruitment in the group and the control group: mean T0-T1 differences

	**Cases**	**Controls**	**p-value**
Frequency of attacks (*)			
Worsening	0 (0%)	0 (0%)	0.005
No change	4 (23.5%)	10 (62.5%)	
Mild improvement	7 (41.2%)	6 (37.5%)	
Moderate improvement	3 (17.6%)	0 (0%)	
Marked improvement	3 (17.6%)	0 (0%)	
Duration of attacks (*)			
Worsening (*)	0 (0%)	0 (0%)	0.002
No change	5 (29.4%)	15 (93.8%)	
Mild improvement	8 (47.1%)	0 (0%)	
Moderate improvement	3 (17.6%)	1 (6.3%)	
Marked improvement	1 (5.9%)	0 (0%)	
Intensity of attacks (*)			
Worsening	0 (0%)	1 (6.3%)	0.001
No change	0 (0%)	9 (56.3%)	
Mild improvement	9 (52.9%)	6 (37.5%)	
Moderate improvement	4 (23.5%)	0 (0%)	
Marked improvement	4 (23.5%)	0 (0%)	

(*)See text for explanation.

**Table 4:** Effects of experimental treatment (vs. controls) on the main headache characteristics after inclusion of drop-outs (intention-to-treat analysis)

	**Imputation of missing value:**		
	**Mild improvement**	**Moderate improvement**	**Marked improvement**
Frequency	χ^2^=8.73 p=0.033	χ^2^=5.56 p=0.135	χ^2^=5.56 p=0.135
Intensity	χ^2^=17.94 p=0.001	χ^2^=14.68 p=0.005	χ^2^=14.68 p=0.005
Duration	χ^2^=14.98 p=0.002	χ^2^=14.07 p=0.003	χ^2^=14.07 p=0.003

The psychotherapeutic treatment was associated with a statistically significant improvement in the CGI score (100% vs 71.4%; p = 0.018) – data not shown – and in the Total competences scale of the CBCL (p = 0.019) (Table 2).

Repeated measures ANOVA showed a time-related improvement in the EuroQoL and CBCL Somatic complaints scores in the entire sample (within-groups factors, T0-T1) and a difference in favour of the experimental treatment in the EuroQoL and CBCL Activities scores (between-groups factors) (Table 2).

When comparing the T0-T1 changes in the two experimental subgroups (individual treatment vs family treatment), no significant differences were found in headache characteristics and other clinical parameters (data not shown).

No adverse events or side effects of treatments were reported both in the experimental and the control group.

## Discussion

We studied a small cohort of patients with idiopathic headache and no psychiatric symptoms who underwent a brief cycle of psychodynamic psychotherapy that focused on separation-individuation difficulties, i.e. subthreshold affective traits. When compared to usual care, the experimental treatment had stronger effects on the intensity of headache attacks and the Competence CBCL scores, although the mean values of the CBCL scores were in the normal range. The children and adolescents with idiopathic headache submitted to psychotherapy, whether in an individual or in a family setting, were found to show a significant improvement (vs controls) in all headache characteristics. This favourable course was associated with an improvement in their Total competences score on the parent-rated CBCL. This score, obtained by summing the scores on the Activities, Social and School scales, may reflect a better adaptation of the patient to his/her environment.

Most CBCL scores however did not show a significant differences in their change comparing patients submitted to psychotherapy with controls. This can be explained by a combination of different factors. First, even at average CBCL scores were in the normal range, so that their change was quite limited. Second, CBCL are filled by parents and therefore it is possible that they might be not enough sensitive to changing in subthreshold affective traits [23]. It is also possible that this depends on parental views, even if latent, regarding the etiopathogenesis of headache (as already shown for mental disorders [24]). Lastly, it is possible that our pilot study was not sufficiently powered to detect differences.

The physician-rated CGI scores also revealed a better post-treatment health status, in the experimental group than in the controls.

Today, the need to evaluate the effectiveness of psychotherapy [25] and at the same time contain health spending are both priority concerns. This pilot study represents an attempt to establish how to verify, applying the principles of evidence-based medicine (e.g. randomization, intention-to-treat analysis), the actual usefulness of a therapeutic approach often applied to young headache patients in clinical practice in our country. The results obtained indicate that a short-term psychodynamic psychotherapeutic approach may be more effective than the usual approach (periodic outpatient visits) in treating childhood and adolescent headache, while also improving dysfunctional emotional and relational mechanisms [26] possibly connected, in a psychosomatic framework, with the pathogenesis of the disease [27].

A Cochrane review found strong evidence for the efficacy of psychological treatment in headache pain reduction in children and adolescents as an outcome of Cognitive-Behavioural Treatment (CBT), Relaxation Training (RT) and biofeedback treatment [28]. A six-fold higher probability of clinically significant improvement in headache was seen with these psychological interventions compared to control conditions (usually waiting list or “usual care”). It is worth noting, however, that a number of different techniques, not always fully manualized, was included under the definition of CBT and RT. Moreover, the effect was significant only on the number and severity of attacks, while quality of life and other psychological dimensions were seldom explored [29]. Given the characteristics of the psychotherapeutic intervention we used, it is possible that it can be more useful in patients with significant emotional problems, even if rated as subthreshold by commonly used scales such as the CBCL; this should however be confirmed in future larger studies.

The study has strengths and limitation. The major strength is the experimental design. Another strength is the contribution of two institutions having the same educational and scientific approach; this limits any bias in the inclusion and assessment of the patients and also controls the reliability to the method of the psychotherapists working in the two institutions. The major limitation is the short follow-up period. We do not know the long-term benefits of our psychotherapeutic program. A second limitation is the small sample size; the study has insufficient power to detect changes in several secondary outcomes, in MO, MA and TTH separately and, in the experimental group, when comparing individual- to family setting. Our findings are therefore to be read as preliminary in view of possible future studies. A third limitation is the single blinded design, which does not exert a proper control of the positive expectations of the experimental approach in the patients’ and parents’ views. A fourth limitation is the exclusion of several patients (among them, individuals with mild headache varieties) which limits the external validity of our data.

## Conclusions

A brief psychodynamic psychotherapy program may be more effective than usual care in children and adolescents with idiopathic headache. This is relevant both for patients’ treatment and for the best possible organization of neuropsychiatric services directed to these children and adolescents.

Future research should seek to establish the possible involvement of other specific or non-specific factors (e.g. number of sessions, variability of settings), and their relative role in the efficacy of the treatment. The specific sensitivity to psychotherapeutic intervention of different headache categories and different age groups (i.e. children vs adolescents) and the persistence of the effect over a longer follow-up period should also be evaluated.

## Competing interests

The authors declare that they have no competing interests.

## Authors’ contributions

UB and CT conceived the study and coordinated the work done; MF, MiR, MaR and GR recruited patients, administered treatments and/or evaluated patients; EB performed statistical analysis; MC participated to the statistical analysis and helped to draft the manuscript. All authors read and approved the final manuscript.

## References

[B1] Abbass A Lovas D Purdy A Direct diagnosis and management of emotional factors in chronic headache patients Cephalalgia 2008 28 1305 1314 10.1111/j.1468-2982.2008.01680.x 18771494

[B2] Trautmann E Lackschewitz H Kröner-Herwig B Psychological treatment of recurrent headache in children and adolescents–a meta-analysis Cephalalgia 2006 26 1411 1426 10.1111/j.1468-2982.2006.01226.x 17116091

[B3] Arita JH Lin J Pinho RS Minett TS de Souza Vitalle MS Fisberg M Peres MF Vilanova LC Masruha MR Adolescents with chronic migraine commonly exhibit depressive symptoms Acta Neurol Belg 2013 113 1 61 65 10.1007/s13760-012-0135-9 23055110

[B4] Maratos J Wilkinson M Migraine in children: a medical and psychiatric study Cephalalgia 1982 2 179 187 10.1046/j.1468-2982.1982.0204179.x 7159920

[B5] Anttila P Sourander A Metsähonkala L Aromaa M Helenius H Sillanpää M Psychiatric symptoms in children with primary headache J Am Acad Child Adolesc Psychiatry 2004 43 412 419 10.1097/00004583-200404000-00007 15187801

[B6] Larsson B The role of psychological, health-behaviour and medical factors in adolescent headache Dev Med Child Neurol 1988 30 616 625 3229559 10.1111/j.1469-8749.1988.tb04799.x

[B7] Arruda MA Bigal ME Behavioral and emotional symptoms and primary headaches in children: a population-based study Cephalalgia 2012 32 1093 1100 10.1177/0333102412454226 22988005

[B8] Balottin U Poli PF Termine C Molteni S Galli F Psychopathological symptoms in child and adolescent migraine and tension-type headache: a meta-analysis Cephalalgia 2013 33 112 122 10.1177/0333102412468386 23203505

[B9] Alexander F Analysis of the therapeutic factors in psychoanalytic treatment. 1950 Psychoanal Q 2007 76 4 1065 1083 18085003 10.1002/j.2167-4086.2007.tb00293.x

[B10] Lanzi G Balottin U Gamba N Fazzi E Psychological aspects of migraine in childhood Cephalalgia 1983 3 Suppl 1 218 220 6616605 10.1177/03331024830030S136

[B11] Sheftell FD Atlas SJ Migraine and psychiatric comorbidity: from theory and hypotheses to clinical application Headache 2002 42 934 944 10.1046/j.1526-4610.2002.02217.x 12390624

[B12] Egger HL Costello EJ Erkanli A Angold A Somatic complaints and psychopathology in children and adolescents: stomach aches, musculoskeletal pains, and headaches J Am Acad Child Adolesc Psychiatry 1999 38 852 860 10.1097/00004583-199907000-00015 10405503

[B13] Leichsenring F Comparative effects of short-term psychodynamic psychotherapy and cognitive-behavioral therapy in depression: a meta-analytic approach Clin Psychol Rev 2001 21 3 401 419 10.1016/S0272-7358(99)00057-4 11288607

[B14] Leichsenring F Rabung S Leibing E The efficacy of short-term psychodynamic psychotherapy in specific psychiatric disorders: a meta-analysis Arch Gen Psychiatry 2004 61 1208 1216 10.1001/archpsyc.61.12.1208 15583112

[B15] Marty P M’uzan M David C L’investigation psycosomatique 1983 Paris: PUF

[B16] Sperling M A further contribution to the psycho-analytic study of migraine and psychogenic headaches. The relation of migraine to depression, states of withdrawal, petit mal, and epilepsy Int J Psychoanal 1964 45 549 557 14236193

[B17] Vincent NF Psychodynamics of a patient with migraine, with a review of the literature Am J Psychother 1960 14 589 605 13842349 10.1176/appi.psychotherapy.1960.14.3.588

[B18] Headache Classification Committee of the International Headache Society Classification and diagnostic criteria for headache disorders, cranial neuralgias and facial pain Cephalalgia 1988 8 Suppl. 7 1 96 3048700

[B19] Guy W ECDEU Assessment Manual for Psychopharmacology - Revised 1976 Rockville MD: Department of Health, Education, and Welfare, Public Health Service, Alcohol, Drug Abuse, and Mental Health Administration, NIMH Psycopharmacology Research Branch, Division of Extramural Research Programs

[B20] The EuroQol Group EuroQol–a new facility for the measurement of health-related quality of life Health Policy 1990 16 3 199 208 10109801 10.1016/0168-8510(90)90421-9

[B21] Achenbach TM Manual for the Child Behaviour Checklist 4–18 and 1991 Profile 1991 Burlington VT: University of Vermont - Department of Psychiatry

[B22] Aliprandi M Pelanda E Senise T Psicoterapia breve di individuazione 1990 Feltrinelli: Milan

[B23] Chiappedi M Maffioletti E Piazza F D’Adda N Tamburini M Balottin U Abilities of preschoolers: comparing different tools Ital J Pediatr 2012 38 3 10.1186/1824-7288-38-3 22281207 PMC3398339

[B24] Mannarini S Boffo M Assessing mental disorder causal beliefs: a latent dimension identification Community Ment Health J 2013 49 6 686 693 10.1007/s10597-012-9581-3 23292323

[B25] Giles TR Prial EM Neims DM Evaluating psychotherapies: a comparison of effectiveness Int J Ment Health 1993 22 43 65

[B26] Roth A Fonagy P What Works for Whom? A Critical Review of Treatments for Children and Adolescents 1997 New York: The Guilford Press

[B27] Balottin U Chiappedi M Rossi M Termine C Nappi G Childhood and adolescent migraine: a neuropsychiatric disorder? Med Hypotheses 2011 76 6 778 781 10.1016/j.mehy.2011.02.016 21356578

[B28] Eccleston C Palermo TM de C Williams AC Lewandowski A Morley S Fisher E Law E Psychological therapies for the management of chronic and recurrent pain in children and adolescents Cochrane Database Syst Rev 2012 12 CD003968 10.1002/14651858.CD003968.pub3 PMC3715398 23235601

[B29] Sieberg CB Huguet A von Baeyer CL Seshia S Psychological interventions for headache in children and adolescents Can J Neurol Sci 2012 39 1 26 34 22384492 10.1017/s0317167100012646

